# The association between maternal factors and milk hormone concentrations: a systematic review

**DOI:** 10.3389/fnut.2024.1390232

**Published:** 2024-07-03

**Authors:** Raabiah Qureshi, Mary Fewtrell, Jonathan C. K. Wells, Sarah Dib

**Affiliations:** UCL Great Ormond Street Institute of Child Health, London, United Kingdom

**Keywords:** breast milk, maternal factors, breast milk hormones, parent-offspring signaling, breast milk composition

## Abstract

**Background:**

Breast milk is the gold standard for infant feeding. It is a dynamic biological fluid rich in numerous bioactive components. Emerging research suggests that these components, including hormones, may serve as signals between mother and offspring. From an evolutionary perspective, maternal hormonal signals could allow co-adaptation of maternal and offspring phenotype, with implications for their Darwinian fitness. However, a series of steps need to be considered to establish the role of a component as a signal and this systematic review focuses on one step: ‘Do maternal factors influence the concentration of milk hormones?’

**Objective:**

To systematically review human studies which analyze the association between maternal factors and the concentration of hormones in breast milk.

**Methods:**

Three databases were searched for studies reporting the association of maternal factors including body mass index (BMI), weight, fat mass, age, ethnicity, smoking with hormones such as adiponectin, leptin, insulin, ghrelin, and cortisol in breast milk.

**Results:**

Thirty-three studies were eligible for inclusion. Maternal BMI was positively associated with milk leptin (20/21 studies) and with milk insulin (4/6 studies). Maternal weight also displayed a positive correlation with milk leptin levels, and maternal diabetes status was positively associated with milk insulin concentrations. Conversely, evidence for associations between maternal fat mass, smoking, ethnicity and other maternal factors and hormone levels in breast milk was inconclusive or lacking.

**Conclusion:**

Current evidence is consistent with a signaling role for leptin and insulin in breast milk, however other steps need to be investigated to understand the role of these components as definitive signals. This review represents a first step in establishing the role of signaling components in human milk and highlights other issues that need to be considered going forward.

## Introduction

The World Health Organisation (WHO) recognizes breast milk as the gold standard for infant feeding. It offers evident short-term advantages for infants, including a decrease in the incidence of mortality and morbidity related to infectious diseases (1). However, breast-feeding is also a mode of nutrition that exhibits substantial variability among mothers, for example in the volume and composition of the milk produced (2).

In this context, breast milk can be considered a medium through which the mother can communicate through different biological pathways with her offspring, potentially regulating the offspring’s growth and development. From an evolutionary perspective, a mother could optimize her Darwinian fitness if her investment in lactation can adapt in response to ongoing environmental factors, whether these relate directly to the mother (e.g., her energy reserves, which support lactation), or to external factors such as the supply of food (e.g., famine), or to psychosocial stress, which could divert maternal metabolic resources to the stress response, and thus reduce nutritional supply to the offspring. Beyond transferring macronutrients and micronutrients to the offspring, other biological molecules could influence how the offspring utilizes its nutritional supply, and hence its developmental trajectory. However, the volume and composition (including hormone content) of breast milk that maximizes maternal Darwinian fitness is not the same that maximizes the offspring’s fitness (3). The offspring may also influence maternal biology, for example through the strength of the suckling response.

Therefore, breastfeeding can be viewed as a dynamic process which involves complex physiological and psychosocial signaling or communication between the mother and the offspring (4).

Human milk contains numerous components that could act as signals between the mother and offspring including hormones, bacteria, nutrients, and growth factors. These components may interact with the infant’s cells, tissues, and organs, triggering various signaling pathways and physiological responses. Among the underlying mechanisms could be epigenetic modifications, including DNA methylation, histone modification, and microRNA effects, and impacts on the establishment of the infant microbiome (5). There are various steps that apply to any component being considered as a signal in milk such as the origin of milk components, whether they come from the mother’s circulation or are synthesized in the breast (or both), if the milk components reach the infant intestine or if they have specific gut receptors. Additional steps include if the milk components are absorbed and influence infant outcomes and whether maternal or environmental factors influence the concentration of milk components. This systematic review focuses on whether maternal and environmental factors influence the concentration of one group of milk components – milk hormones.

Previously, Andreas et al. (6) conducted a systematic review which indicated that there was an association between the concentration of leptin in breast milk and maternal BMI in ten out of fifteen studies (6). Furthermore, a narrative review undertaken in 2016 highlighted reported evidence to support the role of specific bioactive components and explained that several maternal factors such as BMI have been proposed to influence levels of these bioactive components in breast milk (7). In this review, leptin provided the clearest indication that maternal BMI was positively associated with leptin concentrations in breast milk.

The purpose of this review was to systematically search the literature for evidence on maternal and environmental factors that influence the concentration of hormones in human milk.

## Methods

### Eligibility criteria

#### Types of studies

Observational studies and randomized controlled trials (RCTs) that reported on the association between maternal factors and breast milk hormones were eligible for inclusion. Data from RCTs were included only if they reported associations between an exposure (maternal factors) and subsequent measures of milk hormones. Full text studies published in English were included. Studies reported only in abstract form were omitted.

#### Types of participants

Eligible participants included human mother-infant pairs. Studies in which most (>50%) of the infants were exclusively or predominantly breast-fed at the time of sampling were eligible in order to isolate the potential association of maternal factors on breast milk composition. There were no restrictions on participant health status; this was due to the research interest being in how mothers signal their condition and experiences to their offspring, including markers of living conditions, lifestyle, ill health, and good health. Studies in animals were excluded.

### Maternal factors (exposure)

The exposure was any maternal factor related to the mother’s condition, health, lifestyle, living condition or environment at any time period. It was expected that this would include factors such as maternal anthropometry, adiposity, gestational diabetes, age, ethnicity, socioeconomic status, stress, smoking or climate. Any time period was chosen because of the potential of the factors influencing outcomes via various mechanisms such as epigenetic programming and regulation of gene expression which can potentially affect human milk composition.

### Breast milk hormones (outcomes)

Studies were considered eligible if they reported on at least one breast milk hormone. It was expected that this would include cortisol, leptin, insulin, ghrelin, adiponectin, prolactin, oxytocin or resistin. Studies analyzing these hormones in colostrum, transitional or mature milk at any time-point were considered eligible.

### Information sources

Studies were identified by searching electronic databases with no limits on date of publication. The electronic databases searched were MEDLINE Ovid (from 1946), EMBASE Ovid (from 1974) and Cumulative Index of Nursing and Allied Health Literature (CINAHL).

### Search strategy

The search strategy included database-specific search terms and medical subject headings (MeSH) terms with Boolean operators (NOT, AND, OR) were used on synonyms and variations of the terms relating to human milk, hormones, and maternal factors. The search terms were: “breast milk” OR “breastmilk” OR “human milk” OR “breastfeeding” OR “lactation” OR “breastfed” OR “breastfeed” OR “breast fed” OR “breast feed” OR “milk, human” OR “breast feeding” AND “hormones” OR “hormone concentrations” OR “hormonal concentrations” OR “hormone profile” OR “hormones profile” OR “hormone” OR “leptin” OR “insulin” OR “ghrelin” OR “cortisol” OR “prolactin” OR “oxytocin” OR “resistin” OR “adiponectin” OR “thyroid” OR “interleukin-6” OR “tum?r necrosis factor-a” OR “hydrocortisone” AND “parental factors” OR “maternal factors” OR “environmental factors” OR “stress” OR “inflammation” OR “BMI” OR “body mass index” OR “maternal BMI” OR “maternal body mass index” OR “obesity” OR “type 2 diabetes” OR “type 2 diabetes mellitus” OR “T2D” OR “diabetes” OR “diabetes mellitus” OR “university education” OR “weight” OR “height” OR “maternal weight” OR “maternal height.” Searches were limited to human studies. The search was run on 19th June 2023. An example search of MEDLINE can be found in Appendix A.

### Selection strategy

Duplications were removed and references were imported to Covidence for screening according to the eligibility criteria. Titles were screened by RQ, then abstracts and full-texts were screened by two authors independently (RQ and SD). Any discrepancies between the reviewers were discussed to reach consensus.

### Data extraction

Data to be extracted was agreed by the research team. One reviewer (RQ) independently extracted the data from each study. Extracted data included author name, date of publication, sample size, location of study, study design, feeding type and duration, human milk hormones assessed, stage and type of milk analyzed, how the milk sample was processed for analysis, how the samples were obtained, the maternal factors assessed, results of associations, correlation coefficients and confounders adjusted.

### Quality assessment

One independent reviewer (RQ) used the revised Downs and Black Quality Index score system (Appendix B), known to be a reliable and valid tool for assessing bias in observational and randomized studies to assess the quality of individual publications (8). The quality assessment tool provided an overall score based on four assessed domains; reporting, external validity, internal validity bias and internal validity confounding. Each domain had an overall total score out of 10, 3, 7 and 6, respectively. Item 27 relating to the statistical power was given a score of 1when a power analysis had been conducted. Thus, the highest possible score for the checklist was 28 (9). The quality assessment can be found in Appendix C.

### Data synthesis

It was not possible to conduct a meta-analysis on the relationship between maternal factors and human milk composition due to highly heterogenous data for both maternal factors and the hormonal composition of human milk. As a result, the data was synthesized narratively and presented in tables.

## Results

**Figure 1:**
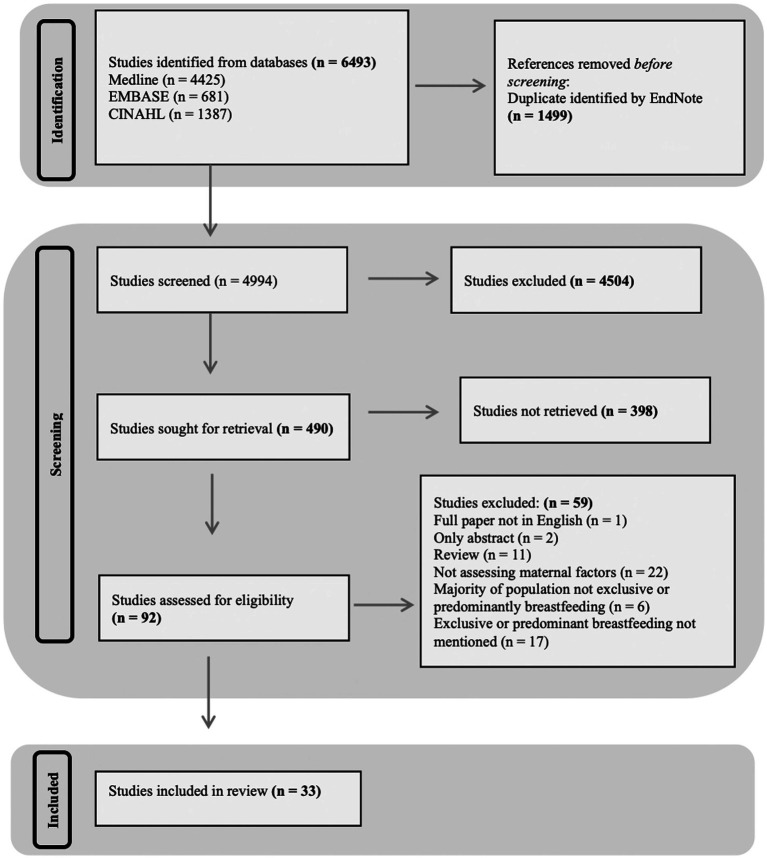
Flow diagram summarizing the process of study screening.

The database searches identified 6,493 studies, of which 4,994 titles were screened following the removal of duplicates. Four hundred ninety abstracts were screened for appropriateness; 398 studies were excluded based on inappropriate title. The full text of 92 studies were reviewed; 59 studies were excluded after the review. This left 33 studies suitable for inclusion in the systematic review. A Preferred Reporting for Systematic Reviews and Meta-Analysis (PRISMA) flow diagram is presented in Figure 1.

Study characteristics are summarized in Table 1. All included studies were conducted in high-income countries with the exception of two conducted in Turkey (11, 35) and one in Iran (20), both classified as upper-middle-income economies. The majority of studies were published in the last 8 years (*n* = 29), except for 5 studies published in 2002, 2006, 2007, 2011 and 2014 (10, 25, 34–36). The main type of study design was observational (*n* = 29), with 4 studies consisting of data from randomized controlled trials (10, 13, 18, 24). Most of the studies had a sample size of 100 or less (*n* = 26) with the largest study including 767 participants (36).

**Table 1:** Summary of the characteristics of included studies.

Paper	Size	Location	Design	HM hormone	Milk type	Analysis of milk	Collection time	Collection method	Maternal factors assessed
Brunner et al. (10)	208	UK	RCT	Adiponectin, Leptin	Mature	Whole milk	After overnight fast	Full breast expression (EP)	BMI, Fat Mass
Cagiran-Yilmaz et al. (11)	65	Turkey	Observational	Leptin	Mature	NS	2 h after feed	Single breast expression (EP)	Weight, BMI, Hip, Waist & Mid-Upper arm circumference
Chan et al. (12)	430	Canada	Observational	Adiponectin, Leptin, Insulin	Mature (foremilk & hindmilk)	Skim Milk	Multiple feeds during 24 h	HE, if unable to do so EP	BMI, Ethnicity, Age, Smoking
Choi et al. (13)	189	USA	RCT	Adiponectin, Leptin, Insulin	Mature	Skim Milk	10 am-12 pm, 2 h post-feed	Single breast expression (EP) post-feed	GDM
Christensen et al. (14)	223	Denmark	Observational	Adiponectin, Leptin, Insulin	Colostrum, Mature	Whole milk	Colostrum – 24-72 h after birth, Human milk – 9 am or 2 pm	Colostrum – HE, Human Milk – Full breast expression (EP)	BMI
Cortes-Macias et al. (15)	100	Spain	Observational	Adiponectin, Leptin	Mature	NS	NS	NS	BMI
De-Luca et al. (16)	100	France	Observational	Leptin	Mature	NS	9 am-11 am during feed	EP	BMI
Ellsworth et al. (17)	55	USA	Observational	Insulin	Mature	Whole milk	8 am-10 am 2 h post-feed	Single breast expression (HE/EP) post-feed	Weight
Enstad et al. (18)	40	USA	RCT	Leptin	Mature	Whole milk	NS	NS	BMI
Fields et al. (7)	37	USA	Observational	Leptin, Insulin	Mature	NS	NS	Single breast expression (EP)	BMI
Gridneva et al. (19)	20	Australia	Obersvational	Adiponectin, Leptin	Mature	Whole milk	NS	NS	Maternal Body Composition
Khodabakshi et al. (20)	80	Iran	Observational	Adiponectin, Leptin, Ghrelin	Mature	NS	8 am-10 am after an overnight fast 2 h post-feed	Full breast expression (EP) post-feed	% Maternal Fat, BMI
Kunganathan et al. (21)	59	Australia	Observational	Adiponectin, Leptin	Mature	Whole & Skim milk	9.30–11.30 am	HE/EP	BMI, % fat mass
Larson-Meyer et al. (22)	22	USA	Observational	Leptin	Mature (foremilk, hindmilk)	NS	7 am-10 am after an overnight fast	Own pump/Mechanical pump	Body mass, fat mass, BMI
Larsson et al. (23)	30	Denmark	Observational	Leptin	Mature (foremilk, hindmilk)	NS	NS	Manual breast pump	BMI
Leghi et al. (24)	15	Australia	RCT	Adiponectin, Leptin, Insulin	Mature	Whole milk	NS	Full breast expression (HE/EP) pre- and post-feed	Body weight, fat mass, carbohydrate and energy intake
Miralles et al. (25)	28	Spain	Observational	Leptin	Mature	NS	9 am-10 am after a 12 h fast	HE	BMI
Nuss et al. (26)	33	USA	Observational	Leptin	Mature	NS	In the morning after a 2 h fast	Single breast expression (EP)	BMI
Pundir et al. (27)	18	Australia	Observational	Cortisol	Mature	NS	9.30 am-10.30 am	Pre- and post-feed	Height, BMI, % fat mass
Pundir et al. (28)	650	Finland	Observational	Cortisol	Mature	NS	Morning	Single breast expression (HE)	Weight, age, GDM, mode of delivery
Rodel et al. (29)	36	USA	Observational	Insulin	Mature	NS	Fasting and mid-feed	HE/EP	Type 2 Diabetes, GDM, Normal glucose tolerance
Romijn et al. (30)	63	Netherlands	Observational	Cortisol	Mature	NS	NS	HE/EP pre-feed	Psychological stress
SadrDadres et al. (31)	135	USA	Observational	Adiponectin, Leptin, Insulin	Mature	Skim milk	8 am-10 am	Single breast expression (EP) post-feed	BMI, gestational weight gain, postpartum weight loss
Savino et al. (32)	58	Italy	Observational	Leptin	Mature (foremilk)	Skim milk	7 am-9 am	HE	BMI
Schneider-Worthington et al. (33)	25	USA	Observational	Adiponectin, Leptin, Insulin	Mature	Skim milk	8 am-10 am following an overnight fast	Personal breast pump	Fat mass
Schuster et al. (34)	23	Germany	Observational	Leptin	Colostrum, Transitional, Mature	Skim milk	NS	NS	BMI
Uysal et al. (35)	50	Turkey	Observational	Leptin	Mature	NS	8 am-11 am	EP post-feed	BMI
Weyermann et al. (36)	767	Germany	Observational	Adiponectin, Leptin	Mature	Whole milk	NS	Full breast expression (HE/EP) pre-feed	Smoking
Young et al. (37)	41	USA	Observational	Insulin	Mature	Whole and skim milk	Fasted mid-feed	HE	Weight
Young et al. (38)	48	USA	Observational	Adiponectin, Leptin, Insulin	Mature	Skim milk	Mid feed	HE	BMI
Yu et al. (39)	96	China	Observational	Adiponectin, Leptin, Insulin, Ghrelin	Colostrum, Mature (foremilk, hindmilk)	NS	Colostrum – 8 am pre-feed, Mature – 2 pm & 4 pm pre-feed	Single breast expression (EP)	GDM, BMI
Zamanillo et al. (40)	59	Spain	Observational	Adiponectin, Leptin	Mature	NS	9 am-2 pm	Full breast expression, (HE, manual breast pump)	BMI
Zielinkska-Pukos et al. (41)	38	Poland	Observational	Cortisol	Mature	NS	4 time periods: 6 am-12 pm, 12 pm-6 pm, 6 pm-12 am, 12 am-6 am	Pre- and post-feeding (HE/EP)	Age, BMI, postpartum depression score, percieved stress scale

HM, human milk; RCT, randomized controlled trial; EP, electronic pump; HE, hand expressed; BMI, body mass index; GDM, gestational diabetes mellitus; NS, not stated.

Human milk components analyzed included concentrations of adiponectin (*n* = 15), leptin (*n* = 26), insulin (*n* = 12), cortisol (*n* = 4) and ghrelin (*n* = 2). Maternal factors assessed included BMI (*n* = 23), fat mass (*n* = 7), weight (*n* = 6), maternal age (*n* = 3), gestational diabetes (*n* = 4), smoking (*n* = 2) and ‘other’ including hip, waist and mid-upper arm circumference, ethnicity, carbohydrate intake, energy intake, psychological stress, and mode of delivery (*n* = 7). Data for all eligible studies including the methods and analysis of sample collection are summarized in Table 1. Mature milk was the main type of milk sample analyzed (*n* = 33), with three studies also analyzing colostrum and one study analyzing transitional milk.

### Synthesis of results

Twenty-three studies examined the relationship between maternal body mass index (BMI) and human milk composition in exclusively or predominantly breastfed infants. The studies investigated associations between maternal BMI pre-, during- and post-pregnancy and adiponectin, leptin, insulin, cortisol, and ghrelin concentrations in milk as shown in Table 2. Among the ten studies focusing on adiponectin, two demonstrated a positive association at 2 weeks and 1–3 months (38, 39), while one reported a negative association stronger at 1 month than 3 months (39). In 20 studies, leptin concentrations, analyzed in twenty-one studies, generally showed a positive correlation with maternal BMI post-pregnancy with higher levels in overweight and obese mothers. For insulin, six studies indicated a positive correlation with maternal BMI post-pregnancy, particularly strong at 3 months, with higher levels in overweight and obese mothers. Cortisol, analyzed in two studies, exhibited a positive correlation with maternal BMI. Ghrelin concentrations, studied in two investigations, displayed an inverse association in one study, while the other found no significant correlation with maternal BMI post-pregnancy. Only three studies among the twenty-three adjusted for potential confounding factors (12, 14, 32).

**Table 2:** Summary of the association between human milk composition and maternal BMI.

Paper	Feeding type	HM measured time-point	HM component	BMI measure time-point	Reported associations and correlations	Confounders adjusted
Brunner et al. (10)	85% EBF and 15% PBF to 6 weeks; 83% EBF and 17% PBF to 4 months	6 weeks and 4 months	Adiponectin (ng mL^−1^)	6 weeks and 4 months	No significant correlations found with BMI	None
			Leptin (ng/mL)	NS	Strong correlation with pre-pregnancy BMI **(*p* < 0.001, *r* = 0.55)**	
Cagiran-Yilmaz et al. (11)	EBF for 3+ months	1, 3 and 6 months	Leptin (ng/mL)	1, 3 and 6 months	Positive correlation with BMI **(*r* < 0.73 across all months and *p* < 0.05)**	None
Chan et al. (12)	EBF for 3–4 months	3 months	Adiponectin (ng mL^−1^)	Pre-pregnancy	No linear correlation observed with pre-pregnancy BMI (*p* = 0.68), however concentrations lower from obese mothers **(*p* = 0.02)**	Maternal BMI, pre-pregnancy weight, gestational weight gain, mode of infant feeding (exclusively or partially breastfed without formula at 3 months postpartum)
			Leptin (ng mL^−1^)		Strong correlation with pre-pregnancy BMI **(*p* < 0.001, *r* = 0.71)**	
			Insulin (ng mL^−1^)		Strong correlation with pre-pregnancy BMI **(*p* < 0.00, *r* = 0.4)**	
Christensen et al. (14)	EBF for 3+ months	Colostrum – 24-72 h after birth, Mature milk – NS	Adiponectin (ng mL^−1^)	NS	No significant associations with maternal BMI	Infant age and mean-center age
			Leptin (ng/mL)		Positive correlation with BMI **(*p* < 0.01)**	
			Insulin (ng mL^−1^)		Positive correlation with BMI **(*p* < 0.01)**	
Cortes-Macias et al. (15)	EBF for 3+ months	15 days post birth	Adiponectin (ng mL^−1^)	NS	No correlation observed (*p* = 0.28)	None
			Leptin (ng/mL)		Positive correlation with BMI **(*p* < 0.01, *r* = 0.388)**	
De-Luca et al. (16)	EBF for 1 month	1 month	Leptin (ng mL^−1^)	1 month	Positive correlation with BMI **(*p* < 0.01, *r* = 0.33)**, concentration higher in obese mothers	None
Enstad et al. (18)	EBF but duration not stated	1 and 4 months	Leptin (ng/mL)	1 and 4 months	Positive correlation with BMI at both time-points	None
Fields et al. (7)	EBF for 3+ months	1 and 6 months	Leptin (ng/mL)	1 and 6 months	Significant association with maternal BMI **(*p* < 0.0001)**, levels 96.5% higher in overweight mothers vs. normal-weight	None
			Insulin (ng mL^−1^)		225% higher in obese mothers with female infants	
Khodabakshi et al. (20)	EBF for 3+ months	6 months	Adiponectin (ng/mL)	6 months	No significant associations with maternal BMI	None
			Leptin (ng/mL)		Significant association with maternal BMI **(*p* < 0.001, *r* = 0.39)**	
			Ghrelin (ng/mL)		No significant associations with maternal BMI	
Kunganathan et al. (21)	EBF for 2–5 months	One of 4 timepoints: 2, 5, 9 and 12 months	Adiponectin (ng/mL)	One of 4 timepoints: 2, 5, 9 and 12 months	No significant associations with maternal BMI (*p* = 0.17)	None
			Leptin (ng/mL)		Significant association with maternal BMI **(*p* < 0.001)**	
Larson-Meyer et al. (22)	EBF for 3+ months	1, 6 and 12 months	Leptin (ng/mL)	1 and 6 months	Significant association with maternal BMI **(*p* < 0.001, *r* = 0.79)**	None
Larsson et al. (23)	EBF for 3+ months	6 weeks	Leptin (ng/mL)	5 and 9 months	Positive correlation with BMI **(*p* < 0.01)** at 5 months and 9 months **(*p* = 0.0057)**	None
Miralles et al. (25)	EBF for 3+ months	1, 3, 6 and 9 months	Leptin (ng/mL)	1, 3, 6 and 9 months	Positive correlation with BMI **(*p* < 0.01, *r* = 0.33)** at each time-point	None
Nuss et al. (26)	EBF for 2 months	NS	Leptin (ng/mL)	NS	Positive correlation with BMI **(*p* < 0.01)**, levels higher in normal BMI	None
Pundir et al. (27)	EBF for 2 months	2 and/or 5, 9 and 12 months	Cortisol (ng/mL)	2 and/or 5, 9 and 12 months	Significant, positive correlation with maternal BMI **(*p* = 0.009, *r* = 0.33)**	None
SadrDadres et al. (31)	EBF for 3+ months	1 and 3 months	Adiponectin (ng mL^−1^)	1 and 3 months	Negative association with BMI **(*p* = 0.02, β = −0.07)**, weaker at 3 months	None
			Leptin (ng/mL)		Positive association with BMI **(*p* < 0.001, β = 0.49)**	
			Insulin (ng mL^−1^)		Positive association with BMI **(*p* = 0.03, β = 0.14)**, stronger at 3 months	
Savino et al. (32)	EBF for 3+ months	3 months	Leptin (ng/mL)	3 months	Positive correlation with BMI **(*p* = 0.004, *r* = 0.37)**	Infant age and gender
Schuster et al. (34)	EBF for 3+ months	1, 2, 3, 4 weeks and 2, 3, 4, 5, and 6 months	Leptin (ng/mL)	1, 2, 3, 4 weeks and 2, 3, 4, 5, and 6 months	Positive correlation with BMI **(*p* < 0.0001, *r* = 0.3)** over 6 months	None
Uysal et al. (35)	EBF for 3+ months	NS	Leptin (ng/mL)	NS	Significant correlation with maternal BMI	None
Young et al. (38)	EBF for 3+ months	2 weeks and 1, 2, 3 and 4 months	Adiponectin (ng/mL)	2 weeks and 1, 2, 3 and 4 months	Positive association at 2 weeks **(*p* = 0.04, *r* = 0.09)**	None
			Leptin (ng/mL)		Increased levels in overweight mothers **(*p* < 0.001)**	
			Insulin (ng/mL)		Increased levels in overweight mothers **(*p* < 0.03)**	
Yu et al. (39)	EBF for 3+ months	Colostrum – 72 h after birth, Mature – 1 (42 days) and 3 months (90 days)	Adiponectin (ng/mL)	Postnatal days 3, 42 and 90	Positive association with BMI **(*p* = 0.001, β = 0.06)**	None
			Leptin (ng/mL)		Positive association with BMI **(*p* < 0.001, β = 0.16)**	
			Insulin (ng/mL)		Positive association with BMI **(*p* < 0.001, β = 0.06)**	
			Ghrelin (ng/mL)		Inversely associated with BMI **(*p* < 0.001, β = −0.08)**	
Zamanillo et al. (40)	86% EBF to 1 month, 85% EBF to 2 months and 78% EBF to 3 months	1, 2 and 3 months	Adiponectin (ng/mL)	1, 2 and 3 months	No associations observed, however, normal weight mothers showed a decrease over time **(*p* < 0.05)**	None
			Leptin (ng/mL)		Negative correlation in normal weight mothers **(*p* < 0.05)**	
Zielinkska-Pukos et al. (41)	EBF for 3+ months	1, 3 and 6 months	Cortisol (ng/mL)	1, 3 and 6 months	No significant associations with maternal BMI	None

Where confounders were adjusted, data reported is from adjusted analysis. Significant results are depicted with the *p* value in bold. HM, human milk; EBF, exclusive breastfeeding; PBF, predominantly breastfeeding; BMI, Body Mass Index; NS, Not Stated.

Seven studies examined the relationship between maternal fat mass and human milk composition, focusing on adiponectin (*n* = 5), leptin (*n* = 6), insulin (*n* = 2), cortisol (*n* = 1), and ghrelin (*n* = 1) as summarized in Table 3. Notably, none of the studies adjusted for potential confounding factors. Regarding adiponectin, only one study reported a significant negative association with fat mass at 6 months (20), while the 4 others found no significant correlations. In the case of leptin, all six studies identified significant associations, with three indicating a positive link between maternal fat mass and leptin concentrations (20, 21, 33). For insulin, one study reported a positive association, while another found no correlation (17). The sole study on cortisol showed no association with maternal fat mass, and the only study on ghrelin also found no correlation.

**Table 3:** Summary of the association between human milk composition and maternal fat mass.

Paper	Feeding type	HM measured time-point	HM component	Maternal fat mass measure time-point	Reported associations and correlations	Confounders adjusted
Brunner et al. (10)	85% EBF and 15% PBF to 6 weeks; 83% EBF and 17% PBF to 4 months	6 weeks and 4 months	Adiponectin (ng mL^−1^)	6 weeks and 4 months	No significant correlations found with fat mass	None
			Leptin (ng/mL)	15th and 32nd week gestation, 6 weeks and 4 months post-partum	Strong correlation with fat mass **(*p* < 0.001)**	
Khodabakshi et al. (20)	EBF for 3+ months	6 months	Adiponectin (ng/mL)	6 months	Significant, negative association with fat mass **(*p* < 0.05, *r* = −0.24)**	None
			Leptin (ng/mL)		Significant, positive association with fat mass **(*p* < 0.01, *r* = 0.39)**	
			Ghrelin (ng/mL)		No significant associations observed with fat mass	
Kunganathan et al. (21)	EBF for 2–5 months	One of 4 timepoints: 2, 5, 9 and 12 months	Adiponectin (ng/mL)	One of 4 timepoints: 2, 5, 9 and 12 months	No significant associations with maternal BMI (*p* = 0.81)	None
			Leptin (ng/mL)		Significant, positive association with fat mass **(*p* = 0.008)**	
Larson-Meyer et al. (22)	EBF for 3+ months	1, 6 and 12 months	Leptin (ng/mL)	1 and 6 months	Significant association with fat mass **(*p* < 0.001, *r* = 0.83)**	None
Leghi et al. (24)	EBF for 6 to 20 weeks	NS	Adiponectin (ng mL^−1^)	NS	No associations observed with fat mass	None
			Leptin (ng/mL)		Significant association with fat mass **(*p* < 0.001)**	
			Insulin (ng/mL)		No associations observed with fat mass	
Pundir et al. (27)	EBF for 2 months	2 and/or 5, 9 and 12 months	Cortisol (ng/mL)	2 and/or 5, 9 and 12 months	No correlation with fat mass (*p* = 0.09)	None
Scheider-Worthington et al. (33)	EBF for 3+ months	NS	Adiponectin (ng mL^−1^)	NS	No significant associations observed with fat mass (*p* = 0.65)	None
			Leptin (ng/mL)		Significant, positive association with fat mass **(*p* < 0.001)**	
			Insulin (ng/mL)		Significant, positive association with fat mass **(*p* = 0.05)**	

Where confounders were adjusted, data is reported from adjusted analysis. Significant results are depicted with the *p* value in bold. HM, human milk; EBF, exclusively breastfeeding; PBF, predominantly breastfeeding; BMI, body mass index; NS, not stated.

Six studies explored the relationship between maternal weight and human milk composition in predominantly breastfed infants, focusing on adiponectin (*n* = 2), leptin (*n* = 3), insulin (*n* = 4), and cortisol (*n* = 1), as detailed in Table 4. Only three studies accounted for potential confounding factors (17, 28, 33). Concerning adiponectin, the two available studies found no correlation with maternal weight. Regarding leptin, all three studies identified significant associations. For insulin, two out of four studies reported a significant association between maternal weight and insulin levels, with one study noting elevated insulin levels in overweight mothers (17). The lone study on cortisol found that normal-weight mothers had higher levels of milk cortisol than mothers with overweight/obesity.

**Table 4:** Summary of the association between human milk composition and maternal weight.

Paper	Feeding type	HM measured time-point	HM component	Maternal weight measure time-point	Reported associations and correlations	Confounders adjusted
Cagiran-Yilmaz et al. (11)	EBF for 3+ months	1, 3 and 6 months	Leptin (ng/mL)	1, 3 and 6 months	Significant, positive correlation with weight **(*p* < 0.01)**	None
Ellsworth et al. (17)	EBF for 3+ months	Day 16 post-partum	Insulin (ng/mL)	Day 16 post-partum	Significant association with weight **(*p* = 0.02)**, higher levels in OW/OB	Infant sex and feeding type (milk only or milk + formula), maternal weight status, pre-pregnancy BMI
Leghi et al. (24)	EBF for 6 to 20 weeks	NS	Adiponectin (ng mL^−1^)	NS	No association observed	None
			Leptin (ng/mL)		Significant, positive association with body weight **(*p* < 0.001)**	
			Insulin (ng/mL)		No association observed	
Pundir et al. (28)	EBF for 3+ months	3 months	Cortisol (ng/mL)	3 months	Significant, positive association with normal weight mothers **(*p* = 0.01)**	Maternal BMI, education, occupational class, family income, family structure, seasonal variation, maternal distress, pregnancy duration, delivery and gestational diabetes
SadrDadres et al. (31)	EBF for 3+ months	1 and 3 months	Adiponectin (ng mL^−1^)	1 and 3 months	No association observed	None
			Leptin (ng/mL)		Positive association with GWG **(*p* < 0.001, β = 0.3)** and negative association with PPWL **(*p* < 0.001, β = −0.18)**	
			Insulin (ng mL^−1^)		No association observed	
Young et al. (37)	EBF for 3+ months	2 weeks and 1, 2, 3 and 4 months	Insulin (ng/mL)	2 weeks and 1, 2, 3 and 4 months	Significant association with weight **(*p* = 0.019)**	Maternal pre-pregnancy BMI group, birth weight, gestational age at delivery, gestational weight gain, maternal age, breastfeeding exclusivity, infant sex and mode of delivery

Where confounders were adjusted, data reported is from adjusted analysis. Significant results are depicted with the *p* value in bold. HM, human milk; EBF, exclusive breastfeeding; PBF, predominantly breastfeeding; BMI, body mass index; GWG, gestational weight gain; PPWL, post-partum weight loss; OW, overweight; OB, obesity; NS, not stated.

Three studies investigated the relationship between maternal age and human milk composition, specifically examining adiponectin (*n* = 1), leptin (*n* = 2), insulin (*n* = 1), and cortisol (*n* = 2), as shown in Table 5. Only two studies accounted for potential confounding factors (12, 28). Regarding adiponectin, a single study found no correlation between maternal age and adiponectin concentrations in human milk (12). In the case of leptin, one study revealed that the concentration of leptin was lower in older women. For insulin, a lone study found no correlation between maternal age and insulin levels in human milk. Two studies explored the association between maternal age and cortisol, with both studies observing no correlation (28, 41).

**Table 5:** Summary of the association between human milk composition and maternal age.

Paper	Feeding type	HM measured time-point	HM component	Maternal age measure time-point	Reported associations and correlations	Confounders adjusted
Chan et al. (12)	EBF for 3–4 months	3 months	Adiponectin (ng mL^−1^)	3 months	No significant correlation observed	Maternal BMI, pre-pregnancy weight, gestational weight gain, mode of infant feeding (exclusively or partially breastfed without formula at 3 months postpartum)
			Leptin (ng mL^−1^)		Concentrations were lower in older women (*p* = 0.05)	
			Insulin (ng mL^−1^)		No significant correlation observed	
Pundir et al. (28)	EBF for 3+ months	3 months	Cortisol (ng/mL)	3 months	No significant effect observed	Maternal BMI, education, occupational class, family income, family structure, seasonal variation, maternal distress, pregnancy duration, delivery and gestational diabetes
Zielinkska-Pukos et al. (41)	EBF for 3+ months	1, 3 and 6 months	Cortisol (ng/mL)	1, 3 and 6 months	No significant correlation observed	None

Where confounders were adjusted, data reported is from adjusted analysis. Significant results are depicted with the *p* value in bold. HM, human milk; EBF, exclusive breastfeeding; BMI, body mass index.

Four studies investigated the link between pre-gestational and gestational maternal diabetes and human milk composition, examining adiponectin (*n* = 2), leptin (*n* = 2), insulin (*n* = 3), cortisol (*n* = 1), and ghrelin (*n* = 1), as detailed in Table 6. Notably, only two studies adjusted for potential confounding factors (13, 28). Regarding adiponectin, one study found a significant negative association with gestational diabetes mellitus in both colostrum and mature milk, while another study found no association (13, 39). For leptin, both studies observed no correlation with maternal diabetes (13, 39). All three studies exploring insulin concentrations reported significant associations with maternal diabetes, with one study revealing lower insulin levels in mothers with gestational diabetes (13) and another indicating that women with type 2 diabetes mellitus had twice the milk insulin levels compared to those with gestational diabetes and normal glucose tolerance (29). The sole study on cortisol found no correlation between maternal diabetes and cortisol in human milk (28) while the single study on ghrelin reported a significant negative correlation with gestational diabetes in both colostrum and mature milk (39).

**Table 6:** Summary of the association between human milk composition and maternal diabetes.

Paper	Feeding type	HM measured time-point	HM component	Maternal diabetes measure time-point	Reported associations and correlations	Confounders adjusted
Choi et al. (13)	EBF for 3+ months	1 and 3 months	Adiponectin (ng mL^−1^)	1 and 3 months	No association observed	Pre-pregnancy BMI, gestational weight gain and breast feeding status (full, partial or none at 3 months)
			Leptin (ng mL^−1^)		No association observed	
			Insulin (ng mL^−1^)		Significant difference found **(*p* = 0.03 and *p* = 0.003)**, lower levels of insulin in GDM	
Pundir et al. (28)	EBF for 3+ months	3 months	Cortisol (ng/mL)	3 months	No significant association found	Maternal BMI, education, occupational class, family income, family structure, seasonal variation, maternal distress, pregnancy duration, delivery and gestational diabetes
Rodel et al. (29)	EBF but duration not stated	2 weeks	Insulin (ng/mL)	2 weeks	Significant difference found **(*p* < 0.001)**, levels 2 times higher in T2DM compared to GDM & NGT	None
Yu et al. (39)	EBF for 3+ months	Colostrum – 72 h after birth, Mature – 1 (42 days) and 3 months (90 days)	Adiponectin (ng/mL)	Colostrum – 72 h after birth, Mature – 1 (42 days) and 3 months (90 days)	Significant, negative correlation with GDM **(pcolostrum < 0.001; pmature = 0.009)**	None
			Leptin (ng/mL)		No significant association found (*p* > 0.05)	
			Insulin (ng/mL)		Significant, positive concentration **(pcolostrum = 0.047; pmature = 0.02)**	
			Ghrelin (ng/mL)		Significant, negative correlation with GDM **(pcolostrum = 0.011; pmature < 0.001)**	

Where confounders were adjusted, data reported is from adjusted analysis. Significant results are depicted with the *p* value in bold. HM, human milk; EBF, exclusive breastfeeding; T2DM, type 2 diabetes mellitus; GDM, gestational diabetes mellitus; BMI, body mass index.

Two studies investigated the relationship between maternal smoking and human milk composition, focusing on adiponectin (*n* = 2), leptin (*n* = 2), and insulin (*n* = 1), as summarized in Table 7. Both studies adjusted for potential confounding factors. However, no significant associations were observed between maternal smoking and adiponectin, leptin, or insulin. Notably, one of the studies found that levels of adiponectin were higher in the milk of non-smoking mothers, suggesting a potential impact of smoking on this specific milk component (36).

**Table 7:** Summary of the association between human milk composition and maternal smoking.

Paper	Feeding type	HM measured time-point	HM component	Maternal smoking measure time-point	Reported associations and correlations	Confounders adjusted
Chan et al. (12)	EBF for 3–4 months	3 months	Adiponectin (ng mL^−1^)	4 months	No significant association found	Maternal BMI, pre-pregnancy weight, gestational weight gain, mode of infant feeding (exclusively or partially breastfed without formula at 3 months postpartum)
			Leptin (ng mL^−1^)		No significant association found	
			Insulin (ng mL^−1^)		No significant association found	
Weyermann et al. (36)	EBF for 3+ months	33 and 71 days post-partum	Adiponectin (ng mL^−1^)	33 and 71 days post-partum	Levels higher in milk with no smoking (*p* value not stated)	Age, education, nationality, BMI, smoking status of mother
			Leptin (ng mL^−1^)		No association found	

Where confounders were adjusted, data reported is from adjusted analysis. Significant results are depicted with the *p* value in bold. HM, human milk; EBF, exclusive breastfeeding; BMI, body mass index.

Table 8 presents a summary of findings on the associations between the hormonal composition of human milk and other maternal factors. Seven studies explored the relationships between these factors and adiponectin (*n* = 3), leptin (*n* = 4), insulin (*n* = 2), and cortisol (*n* = 3), with two studies adjusting for potential confounding factors. Regarding adiponectin, a study found lower levels in Asian mothers compared to Caucasian mothers (12), while another reported a positive association with changes in carbohydrate and total energy intake (24). However, a study observed no significant association with maternal body composition (19). For leptin, previous research identified positive correlations with hip, waist, and mid-upper arm circumferences at different postpartum time points (11). No associations were observed with ethnicity, maternal body composition, and carbohydrate and energy intake in other studies. In terms of insulin, Chan et al. (12) reported higher levels in Asian mothers compared to Caucasians, while another study found no association with maternal carbohydrate and energy intake (12). For cortisol, Romijn et al. (30) noted lower levels in mothers seeking psychiatric consultation compared to healthy controls, and another study found no association with postpartum depression score or perceived stress (41). Pundir et al. (41) observed no association between mode of delivery and cortisol levels at 3 months.

**Table 8:** Summary of the association between human milk composition and other maternal factors.

Paper	Feeding type	HM measured time-point	HM component	Other maternal factors measure time-point	Reported associations and correlations	Confounders adjusted
Cagiran-Yilmaz et al. (11)	EBF for 3+ months	1, 3 and 6 months	Leptin (ng/mL)	1, 3 and 6 months	Significant, positive correlation with hip, waist and mid-upper arm circumference in all months **(*p* < 0.05)**	None
Chan et al. (12)	EBF for 3–4 months	3 months	Adiponectin (ng mL^−1^)	4 months	Asian mothers had lower levels compared to Caucasian **(*p* = 0.01)**	Maternal BMI, pre-pregnancy weight, gestational weight gain, mode of infant feeding (exclusively or partially breastfed without formula at 3 months postpartum)
			Leptin (ng mL^−1^)		No association observed with ethnicity	
			Insulin (ng mL^−1^)		Asian mothers had higher levels compared to Caucasian **(*p* < 0.0001)**	
Gridneva et al. (19)	EBF for 3+ months	2 and/or 5, 9 and 12 months	Adiponectin (ng mL^−1^)	2 and/or 5, 9 and 12 months	No significant association observed with maternal body composition	None
			Leptin (ng/mL)		No significant association observed with maternal body composition	
Leghi et al. (24)	EBF for 6 to 20 weeks	NS	Adiponectin (ng mL^−1^)	NS	Positive association with changes in maternal carbohydrate intake **(*p* = 0.033)** and total energy intake **(*p* = 0.038)**	None
			Leptin (ng/mL)		No association observed with maternal carbohydrate and energy intake	
			Insulin (ng/mL)		No association observed with maternal carbohydrate and energy intake	
Romijn et al. (30)	EBF for 3+ months	1 month	Cortisol (ng/mL)	1 month	Cortisol was lower in the group who sought consultation at the psychiatry-obstetric-pediatric outpatient clinic **(*p* = 0.02)**	None
Pundir et al. (28)	EBF for 3+ months	3 months	Cortisol (ng/mL)	3 months	No association observed with mode of delivery	Maternal BMI, education, occupational class, family income, family structure, seasonal variation, maternal distress, pregnancy duration, delivery and gestational diabetes
Zielinkska-Pukos et al. (41)	EBF for 3+ months	1, 3 and 6 months	Cortisol (ng/mL)	1, 3 and 6 months	No association observed with postpartum depression score and percieved stress scale	None

Where confounders were adjusted, data reported is from adjusted analysis. Significant results are depicted with the *p* value in bold. HM, human milk; EBF, exclusive breastfeeding; BMI, body mass index; NS, not stated.

## Discussion

**Figure 2:**
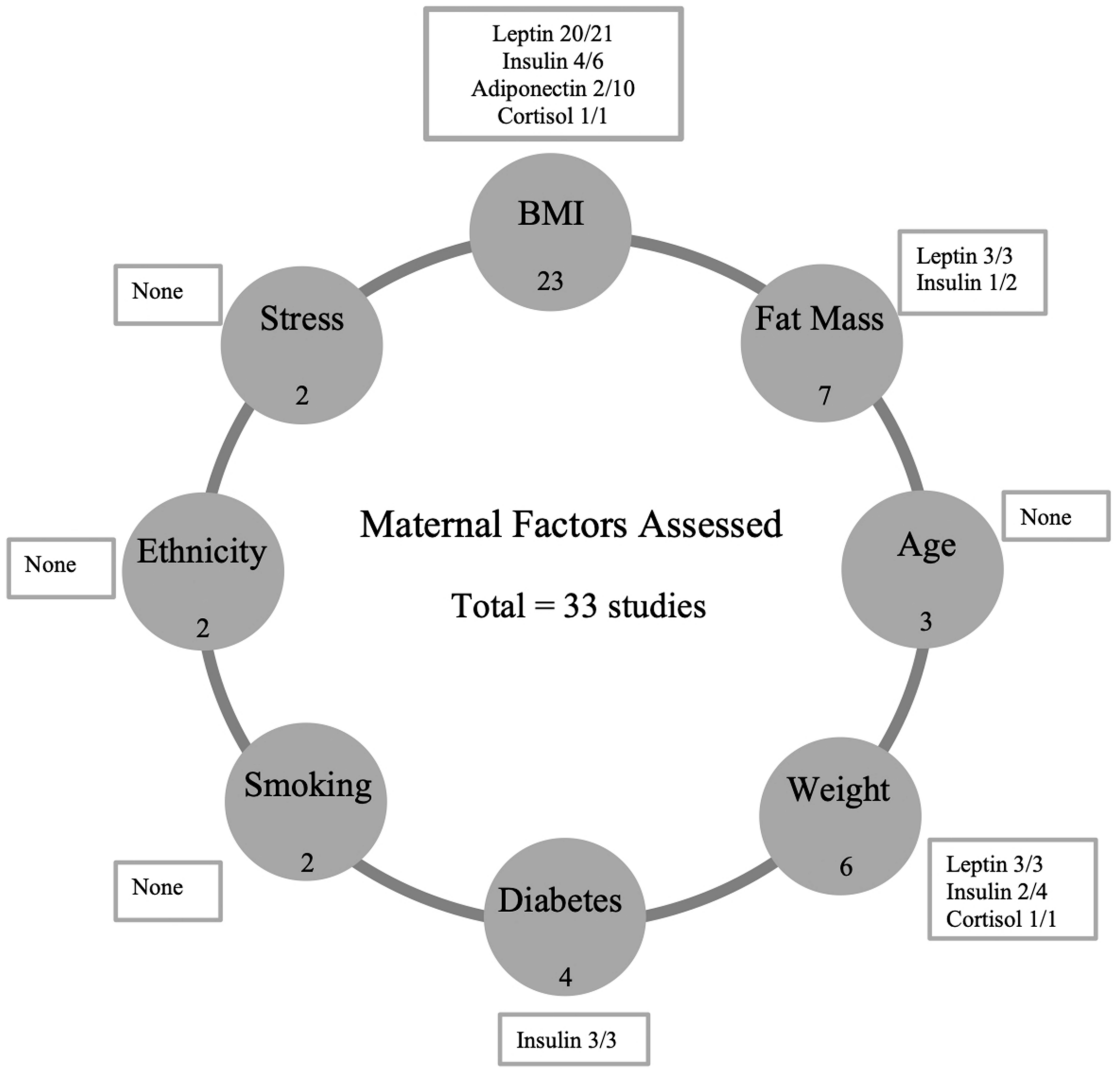
Summary of results. This figure shows the positive associations found between specific maternal factors and the concentration of breast milk hormones in this systematic review. The number of studies included which reported each maternal factor (i.e., BMI -23) are represented in filled circles. The number of studies that found a positive association between the maternal factor and hormone are shown in textboxes (i.e., Leptin 20 studies found a positive association out of a total of 21 studies). BMI, body mass index.

This systematic review explored the relationship between different maternal factors and hormones in breast milk, as a first step to establishing their role in signaling mechanisms between mother and infant. The review of 33 papers suggests a positive association between maternal adiposity (BMI and weight either pre-pregnancy and during lactation) and breast milk leptin concentrations (Figure 2). However, the evidence regarding maternal fat mass, age, smoking, and other factors was inconclusive. The review underscores the need for more research in this area, emphasizing the inconsistency in findings, likely due to variations in data collection and sampling methods across studies.

Maternal adiposity was assessed using a variety of measures in different studies. There was a positive association between maternal BMI and breast milk leptin and insulin in the majority of studies included with only 2/10 studies showing a positive association between maternal BMI and adiponectin. This aligns with a previous systematic review (6) by Andreas et al. (6) which also found an association of maternal BMI with breast milk leptin but not adiponectin (6). A 2016 narrative review further highlighted the positive association between maternal BMI and leptin and insulin levels in human breast milk (7). We also found that maternal weight was positively associated with milk leptin in all included studies, but its relationship with milk insulin was less clear. Inconsistent associations were also observed between maternal fat mass and milk leptin. It is possible that the associations of milk hormones with BMI were stronger than with fat mass due to the ease with which BMI could be assessed and thus the larger sample sizes in studies using this outcome.

Leptin in breast milk could serve as a signaling mechanism for the infant, affecting aspects such as metabolism, appetite, and fat storage. The leptin gene (LEP), which is responsible for the production of leptin, is expressed in mammary epithelial cells and can be influenced by many factors including maternal diet, nutritional status and hormone regulation (42). Since leptin signals the level of fat reserves to the brain, maternal leptin transfer to the infant could act to inflate such signals, effectively manipulating the infant’s brain into over-estimating its fat stores and thus impact its appetite. While studies have indicated that higher leptin in breast milk might indeed reduce infant appetite, this could have both positive and negative implications (32). Positive outcomes include better self-regulation, more appropriate feeding patterns and reduced long term obesity risk, while negatives could be inadequate nutrition and suboptimal growth. Similarly, higher insulin levels in milk were previously found to be associated with lower infant weight and weight-for-length z-scores (14). However, other studies found no link between milk insulin and infant anthropometrics.

This review confirmed that maternal diabetes is associated with significantly higher insulin levels in breast milk. Maternal insulin regulation can be disrupted in type 2 diabetes, leading to increased insulin levels in maternal circulation and potentially influencing its presence in breast milk. A study showed that women with type 2 diabetes had significantly higher insulin levels in their breast milk compared to those with gestational diabetes and normal glucose tolerance, possibly due to insulin therapy and injections (28). Limited research exists on the impact of breast milk constituents on the growth of infants born to mothers with diabetes during pregnancy, especially gestational diabetes. Previous data suggests that breast milk from diabetic mothers may lead to increased relative body weight and obesity at two years of age, while milk from healthy non-diabetic women had a beneficial effect on later body weight and glucose tolerance in childhood (43).

Aging is associated with many changes in the levels of several hormones in maternal plasma which could have an effect on breast milk hormone levels. For example, Isidori et al. (44) indicated that serum leptin gradually declines during aging but is independent from BMI and other age-related endocrine changes. However, only one study included in this review found that the concentration of leptin was lower in older women (12) with the remaining studies reporting no association between milk hormones and maternal age.

The effect of smoking on breast milk hormones might be expected to vary depending on factors such as smoking intensity, duration, and individual differences (45). Research indicates that smoking ten or more cigarettes a day can adversely affect lactation by reducing milk production and altering macronutrient content (45). Smoking during pregnancy and lactation can also impact maternal health, increasing stress and anxiety levels, potentially altering breast milk composition and hormone levels (43). Nonetheless, this review only included two studies on maternal smoking and breast milk hormones, one reporting higher levels of adiponectin in the milk of non-smoking mothers (36), while the other (14) found no association, likely due to low smoking rates in their study population. This highlights the need for further research to comprehensively understand how smoking influences breast milk hormonal composition and its impact on infant outcomes.

Ethnicity could theoretically influence hormone levels in breast milk due to a combination of genetic, cultural, environmental and lifestyle factors that vary among different ethnic groups. However, this review only found one study that mentioned ethnicity in relation to milk hormone levels, concluding that Asian mothers had lower levels of adiponectin and higher levels of insulin in their milk when compared to Caucasian mothers (12). This difference could reflect factors such as body composition as ethnic groups can have distinct body composition characteristics which could impact hormone production and metabolism (46). In addition, cultural dietary practices and certain foods and nutrients vary among different ethnic groups. Diets with anti-inflammatory properties, such as the Mediterranean diet and others emphasizing plant-based foods and healthy fats, have demonstrated the ability to lower leptin levels in circulating blood and enhance leptin sensitivity (47). Conversely, heightened intakes of saturated fatty acids have been linked to inducing leptin resistance by interrupting leptin signaling after chronic overstimulation of the leptin receptor (48). However, research on the specific differences in breast milk composition among various ethnicities is limited.

The review included two studies on post-partum depression and stress in relation to breast milk cortisol levels. One study reported that mothers in a psychiatry-obstetric-pediatric clinic had lower milk cortisol compared to those not in the clinic (30). However, this study had limitations, as it focused on a specific population with a higher risk of psychological distress during pregnancy. On the other hand, another study did not find a significant association between maternal depression and breast milk cortisol (29). Chronic stress can affect the hypothalamic–pituitary–adrenal (HPA) axis, potentially leading to elevated cortisol levels in breast milk. While previous studies have explored the connection between maternal psychological factors and milk cortisol levels, they have yielded inconsistent results. Objective assessments of maternal cortisol levels in plasma appear to provide a more reliable measure of chronic stress compared to subjective methods used in the studies reviewed.

### Mother-infant signaling

The results from this systematic review suggest that the mother may communicate important cues through the hormonal composition of breast milk. The studies consistently revealed the presence of diverse hormones such as adiponectin, leptin, insulin, cortisol, and ghrelin in breast milk. The varying concentration of these hormones (specifically leptin and insulin) in relation to maternal factors such as BMI, weight and other health indicators highlight an intricate interplay between maternal physiology and breast milk composition. This supports the notion that maternal factors are somewhat linked to the hormonal makeup of breast milk in particular maternal BMI and weight, which, in turn, could potentially impact infant outcomes such as growth, development and overall health. Notably, associations between maternal nutritional status and offspring development, mediated by milk hormones, have recently been reported in primates (49). Breast milk hormones could potentially influence infant outcomes through epigenetic mechanisms such as changes in the gene expression that could be triggered by various environmental, nutritional and hormonal factors during pregnancy and lactation (50).

**Figure 3:**
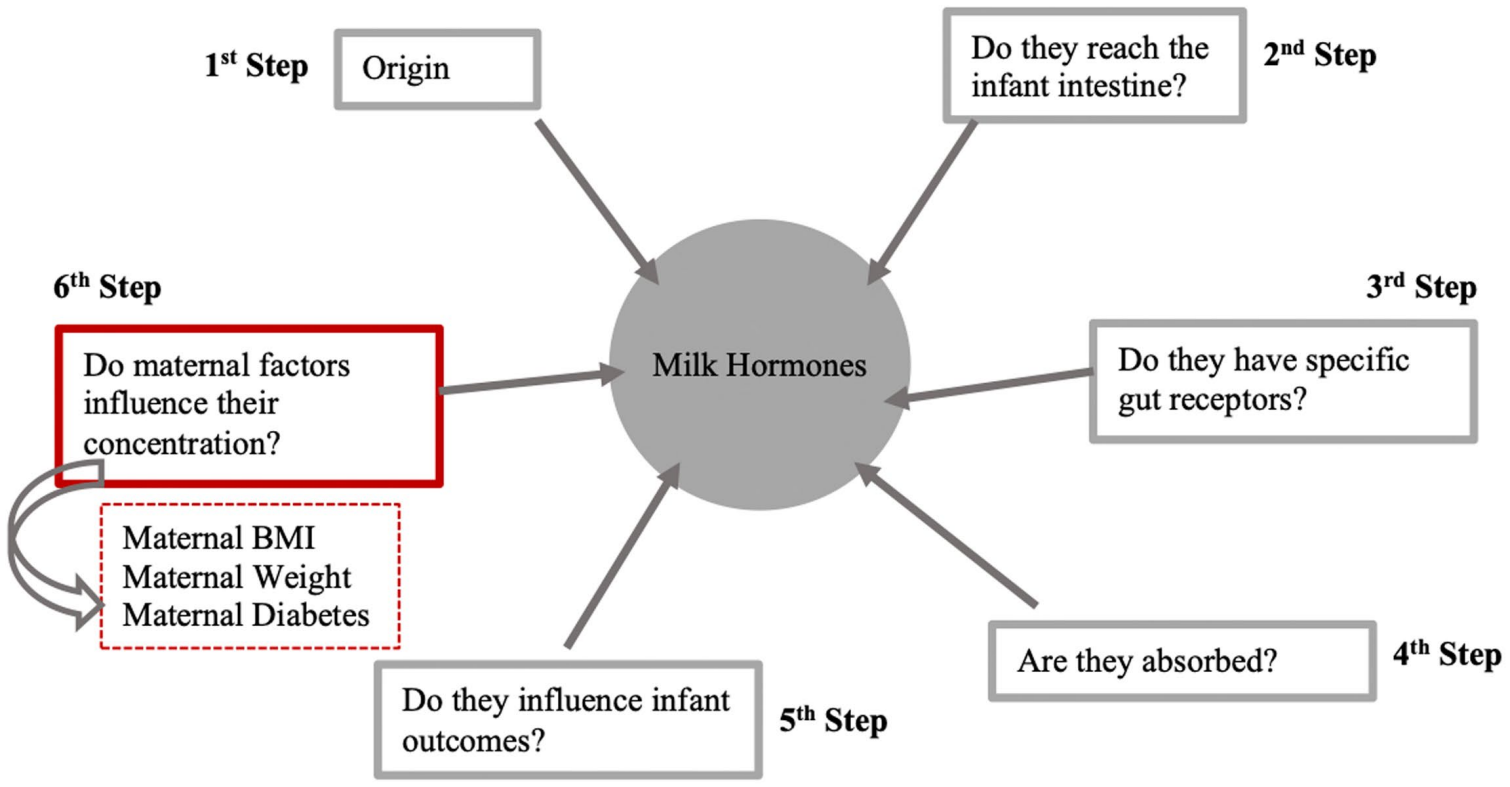
Six steps to determine whether a milk hormone acts as a signal between mother and offspring. This figure summarizes the results from this systematic review which aimed to obtain evidence for the sixth stage of the process. BMI, body mass index. Figure adapted from Fewtrell et al., (4), licensed under CC BY 4.0.

However, there are six steps that should be fulfilled for a component to act as a signal (Figure 3), with the results from this review contributing evidence toward one step only in establishing whether a hormone is a signaling component, by assessing which maternal factors affect their concentration in human breast milk. This review did not find any studies assessing the association between maternal factors and hormones such as prolactin, oxytocin and resistin. The lack of current evidence found does not rule out a signaling role of these hormones. Instead, it highlights a notable gap in the existing literature in relation to other hormones within breast milk. In addition, this review solely focused on hormones as potential signaling components, but there are many other possible signals in milk such as bacteria, nutrients, and growth factors. These other components could be investigated using the same approach as this systematic review.

### Limitations

This systematic review has highlighted limitations stemming from inadequate study design, hindering our understanding of how maternal factors affect the composition of bioactive components in breast milk. The complexity of maternal influences, both genetic and environmental, makes it challenging to draw causal inferences, as randomizing subjects based on many relevant factors is practically impossible. New study designs are needed, such as long-term observational studies that follow mother-infant pairs over time or clustering them by similar characteristics like BMI. Targeted interventions, focusing on factors like maternal diet or stress reduction, can provide insights into how specific changes impact breast milk composition.

Additionally, the variability in sampling methods among different studies, including lactation stage, feeding frequency, and time of day, complicates direct comparisons of findings. Inconsistencies may arise from the lack of standardization in collecting and processing human milk samples. For instance, some hormones show diurnal patterns influenced by the time of day, and variations in sampling techniques, such as analyzing foremilk or hindmilk, can affect results. Notably, the quality of the studies reviewed was generally low or fair, and many studies lacked reporting on maternal factors or breastfeeding patterns, making it difficult to assess the strength of the associations between maternal factors and breast milk hormone concentrations.

## Conclusion

This systematic review suggests that higher maternal BMI is linked to increased breast milk leptin and maternal diabetes to higher breast milk insulin. However, it fails to establish clear associations between maternal fat mass, age, smoking, ethnicity, stress, and breast milk hormonal composition due to insufficient data and methodological limitations in prior studies. The use of standardized protocols for sample collection and analysis in future studies would enable more meaningful cross-study comparisons. Further research is required to understand how breast milk hormones affect infant outcomes and their role as signaling components. In conclusion, this study underscores the complex relationship between maternal factors, breast milk composition, and potential infant signaling mechanisms, serving as a starting point for future investigations.

## Data availability statement

The original contributions presented in the study are included in the article/Supplementary material, further inquiries can be directed to the corresponding author.

## Author contributions

RQ: Writing – original draft. MF: Writing – review & editing. JW: Writing – review & editing. SD: Writing – review & editing.
