# Supplementary material for: The association between maternal factors and milk hormone concentrations: a systematic review
Source: Front Nutr. 2024 Jul 3;11:1390232. doi: 10.3389/fnut.2024.1390232 (PMC11253774; doi:10.3389/fnut.2024.1390232)
Supplement: Supplementary file 1 [file Data_Sheet_1.PDF]

Supplementary material
Appendix

1. Appendix A: MEDLINE Ovid Search run on 19th June 2023

MEDLINE Ovid Search Terms	Records returned
breast milk.mp. [mp=title, book title, abstract, original title, name of substance word, subject heading word, floating sub-heading word, keyword heading word, organism supplementary concept word, protocol supplementary concept word, rare disease supplementary concept word, unique identifier, synonyms, population supplementary concept word, anatomy supplementary concept word]	16299
breastmilk.mp. [mp=title, book title, abstract, original title, name of substance word, subject heading word, floating sub-heading word, keyword heading word, organism supplementary concept word, protocol supplementary concept word, rare disease supplementary concept word, unique identifier, synonyms, population supplementary concept word, anatomy supplementary concept word]	2666
human milk.mp. [mp=title, book title, abstract, original title, name of substance word, subject heading word, floating sub-heading word, keyword heading word, organism supplementary concept word, protocol supplementary concept word, rare disease supplementary concept word, unique identifier, synonyms, population supplementary concept word, anatomy supplementary concept word	13681
breastfeeding.mp. [mp=title, book title, abstract, original title, name of substance word, subject heading word, floating sub-heading word, keyword heading word, organism supplementary concept word, protocol supplementary concept word, rare disease supplementary concept word, unique identifier, synonyms, population supplementary concept word, anatomy supplementary concept word]	35289
lactation.mp. [mp=title, book title, abstract, original title, name of substance word, subject heading word, floating sub-heading word, keyword heading word, organism supplementary concept word, protocol supplementary concept word, rare disease supplementary concept word, unique identifier, synonyms, population supplementary concept word, anatomy supplementary concept word]	69908
breastfed.mp. [mp=title, book title, abstract, original title, name of substance word, subject heading word, floating sub-heading word, keyword heading word, organism supplementary concept word, protocol supplementary concept word, rare disease supplementary concept word, unique identifier, synonyms, population supplementary concept word, anatomy supplementary concept word]	8513
breastfeed.mp. [mp=title, book title, abstract, original title, name of substance word, subject heading word, floating sub-heading word, keyword heading word, organism supplementary concept word, protocol supplementary concept word, rare disease supplementary concept word, unique identifier, synonyms, population supplementary concept word, anatomy supplementary concept word]	3947
breast fed.mp. [mp=title, book title, abstract, original title, name of substance word, subject heading word, floating sub-heading word, keyword heading word, organism supplementary concept word, protocol supplementary concept word, rare disease supplementary concept word, unique identifier, synonyms, population supplementary concept word, anatomy supplementary concept word]	5938

breast feed.mp. [mp=title, book title, abstract, original title, name of substance word, subject heading word, floating sub-heading word, keyword heading word, organism supplementary concept word, protocol supplementary concept word, rare disease supplementary concept word, unique identifier, synonyms, population supplementary concept word, anatomy supplementary concept word]	1230
Milk, Human/	22720
Breast Feeding/	43895
Lactation/	47898
hormones.mp. [mp=title, book title, abstract, original title, name of substance word, subject heading word, floating sub-heading word, keyword heading word, organism supplementary concept word, protocol supplementary concept word, rare disease supplementary concept word, unique identifier, synonyms, population supplementary concept word, anatomy supplementary concept word]	314820
'hormone concentrations'.mp. [mp=title, book title, abstract, original title, name of substance word, subject heading word, floating sub-heading word, keyword heading word, organism supplementary concept word, protocol supplementary concept word, rare disease supplementary concept word, unique identifier, synonyms, population supplementary concept word, anatomy supplementary concept word]	6971
'hormonal concentrations'.mp. [mp=title, book title, abstract, original title, name of substance word, subject heading word, floating sub-heading word, keyword heading word, organism supplementary concept word, protocol supplementary concept word, rare disease supplementary concept word, unique identifier, synonyms, population supplementary concept word, anatomy supplementary concept word]	386
'hormone profile'.mp. [mp=title, book title, abstract, original title, name of substance word, subject heading word, floating sub-heading word, keyword heading word, organism supplementary concept word, protocol supplementary concept word, rare disease supplementary concept word, unique identifier, synonyms, population supplementary concept word, anatomy supplementary concept word]	922
'hormones profile'.mp. [mp=title, book title, abstract, original title, name of substance word, subject heading word, floating sub-heading word, keyword heading word, organism supplementary concept word, protocol supplementary concept word, rare disease supplementary concept word, unique identifier, synonyms, population supplementary concept word, anatomy supplementary concept word]	43
leptin.mp. [mp=title, book title, abstract, original title, name of substance word, subject heading word, floating sub-heading word, keyword heading word, organism supplementary concept word, protocol supplementary concept word, rare disease supplementary concept word, unique identifier, synonyms, population supplementary concept word, anatomy supplementary concept word]	42945
insulin.mp. [mp=title, book title, abstract, original title, name of substance word, subject heading word, floating sub-heading word, keyword heading word, organism supplementary concept word, protocol supplementary concept word, rare disease supplementary concept word, unique identifier, synonyms, population supplementary concept word, anatomy supplementary concept word]	467410
adiponectin.mp. [mp=title, book title, abstract, original title, name of substance word, subject heading word, floating sub-heading word, keyword heading word, organism supplementary concept word, protocol supplementary concept word, rare disease supplementary concept word, unique identifier, synonyms, population supplementary concept word, anatomy supplementary concept word]	25001

ghrelin.mp. [mp=title, book title, abstract, original title, name of substance word, subject heading word, floating sub-heading word, keyword heading word, organism supplementary concept word, protocol supplementary concept word, rare disease supplementary concept word, unique identifier, synonyms, population supplementary concept word, anatomy supplementary concept word]	12403
cortisol.mp. [mp=title, book title, abstract, original title, name of substance word, subject heading word, floating sub-heading word, keyword heading word, organism supplementary concept word, protocol supplementary concept word, rare disease supplementary concept word, unique identifier, synonyms, population supplementary concept word, anatomy supplementary concept word]	70170
oxytocin.mp. [mp=title, book title, abstract, original title, name of substance word, subject heading word, floating sub-heading word, keyword heading word, organism supplementary concept word, protocol supplementary concept word, rare disease supplementary concept word, unique identifier, synonyms, population supplementary concept word, anatomy supplementary concept word]	30729
prolactin.mp. [mp=title, book title, abstract, original title, name of substance word, subject heading word, floating sub-heading word, keyword heading word, organism supplementary concept word, protocol supplementary concept word, rare disease supplementary concept word, unique identifier, synonyms, population supplementary concept word, anatomy supplementary concept word]	54804
thyroid.mp. [mp=title, book title, abstract, original title, name of substance word, subject heading word, floating sub-heading word, keyword heading word, organism supplementary concept word, protocol supplementary concept word, rare disease supplementary concept word, unique identifier, synonyms, population supplementary concept word, anatomy supplementary concept word]	240539
interleukin-6.mp. [mp=title, book title, abstract, original title, name of substance word, subject heading word, floating sub-heading word, keyword heading word, organism supplementary concept word, protocol supplementary concept word, rare disease supplementary concept word, unique identifier, synonyms, population supplementary concept word, anatomy supplementary concept word]	108665
tum?r necrosis factor-a.mp. [mp=title, book title, abstract, original title, name of substance word, subject heading word, floating sub-heading word, keyword heading word, organism supplementary concept word, protocol supplementary concept word, rare disease supplementary concept word, unique identifier, synonyms, population supplementary concept word, anatomy supplementary concept word]	1023
resistin.mp. [mp=title, book title, abstract, original title, name of substance word, subject heading word, floating sub-heading word, keyword heading word, organism supplementary concept word, protocol supplementary concept word, rare disease supplementary concept word, unique identifier, synonyms, population supplementary concept word, anatomy supplementary concept word]	5195
Hormones/	39543
Leptin/	26152
Insulin/	199268
Adiponectin/	14236
Ghrelin/	8399
Hydrocortisone/	77015
Oxytocin/	21598
Prolactin/	40369
Resistin/	2663
parental factors.mp. [mp=title, book title, abstract, original title, name of substance word, subject heading word, floating sub-heading word, keyword	726

heading word, organism supplementary concept word, protocol supplementary concept word, rare disease supplementary concept word, unique identifier, synonyms, population supplementary concept word, anatomy supplementary concept word]	
maternal factors.mp. [mp=title, book title, abstract, original title, name of substance word, subject heading word, floating sub-heading word, keyword heading word, organism supplementary concept word, protocol supplementary concept word, rare disease supplementary concept word, unique identifier, synonyms, population supplementary concept word, anatomy supplementary concept word]	3039
environmental factors.mp. [mp=title, book title, abstract, original title, name of substance word, subject heading word, floating sub-heading word, keyword heading word, organism supplementary concept word, protocol supplementary concept word, rare disease supplementary concept word, unique identifier, synonyms, population supplementary concept word, anatomy supplementary concept word]	83624
stress.mp. [mp=title, book title, abstract, original title, name of substance word, subject heading word, floating sub-heading word, keyword heading word, organism supplementary concept word, protocol supplementary concept word, rare disease supplementary concept word, unique identifier, synonyms, population supplementary concept word, anatomy supplementary concept word]	1163310
inflammation.mp. [mp=title, book title, abstract, original title, name of substance word, subject heading word, floating sub-heading word, keyword heading word, organism supplementary concept word, protocol supplementary concept word, rare disease supplementary concept word, unique identifier, synonyms, population supplementary concept word, anatomy supplementary concept word]	688599
BMI.mp. [mp=title, book title, abstract, original title, name of substance word, subject heading word, floating sub-heading word, keyword heading word, organism supplementary concept word, protocol supplementary concept word, rare disease supplementary concept word, unique identifier, synonyms, population supplementary concept word, anatomy supplementary concept word]	194493
body mass index.mp. [mp=title, book title, abstract, original title, name of substance word, subject heading word, floating sub-heading word, keyword heading word, organism supplementary concept word, protocol supplementary concept word, rare disease supplementary concept word, unique identifier, synonyms, population supplementary concept word, anatomy supplementary concept word]	300263
maternal BMI.mp. [mp=title, book title, abstract, original title, name of substance word, subject heading word, floating sub-heading word, keyword heading word, organism supplementary concept word, protocol supplementary concept word, rare disease supplementary concept word, unique identifier, synonyms, population supplementary concept word, anatomy supplementary concept word]	1747
maternal body mass index.mp. [mp=title, book title, abstract, original title, name of substance word, subject heading word, floating sub-heading word, keyword heading word, organism supplementary concept word, protocol supplementary concept word, rare disease supplementary concept word, unique identifier, synonyms, population supplementary concept word, anatomy supplementary concept word]	1635
obesity.mp. [mp=title, book title, abstract, original title, name of substance word, subject heading word, floating sub-heading word, keyword heading word, organism supplementary concept word, protocol supplementary concept word, rare disease supplementary concept word, unique identifier, synonyms, population supplementary concept word, anatomy supplementary concept word]	405892
maternal obesity.mp. [mp=title, book title, abstract, original title, name of substance word, subject heading word, floating sub-heading word, keyword	3472

heading word, organism supplementary concept word, protocol supplementary concept word, rare disease supplementary concept word, unique identifier, synonyms, population supplementary concept word, anatomy supplementary concept word]	
type 2 diabetes.mp. [mp=title, book title, abstract, original title, name of substance word, subject heading word, floating sub-heading word, keyword heading word, organism supplementary concept word, protocol supplementary concept word, rare disease supplementary concept word, unique identifier, synonyms, population supplementary concept word, anatomy supplementary concept word]	168323
T2D.mp. [mp=title, book title, abstract, original title, name of substance word, subject heading word, floating sub-heading word, keyword heading word, organism supplementary concept word, protocol supplementary concept word, rare disease supplementary concept word, unique identifier, synonyms, population supplementary concept word, anatomy supplementary concept word]	16784
type 2 diabetes mellitus.mp. [mp=title, book title, abstract, original title, name of substance word, subject heading word, floating sub-heading word, keyword heading word, organism supplementary concept word, protocol supplementary concept word, rare disease supplementary concept word, unique identifier, synonyms, population supplementary concept word, anatomy supplementary concept word]	62543
diabetes.mp. [mp=title, book title, abstract, original title, name of substance word, subject heading word, floating sub-heading word, keyword heading word, organism supplementary concept word, protocol supplementary concept word, rare disease supplementary concept word, unique identifier, synonyms, population supplementary concept word, anatomy supplementary concept word]	759846
diabetes mellitus.mp. [mp=title, book title, abstract, original title, name of substance word, subject heading word, floating sub-heading word, keyword heading word, organism supplementary concept word, protocol supplementary concept word, rare disease supplementary concept word, unique identifier, synonyms, population supplementary concept word, anatomy supplementary concept word]	532557
university education.mp. [mp=title, book title, abstract, original title, name of substance word, subject heading word, floating sub-heading word, keyword heading word, organism supplementary concept word, protocol supplementary concept word, rare disease supplementary concept word, unique identifier, synonyms, population supplementary concept word, anatomy supplementary concept word]	1797
weight.mp. [mp=title, book title, abstract, original title, name of substance word, subject heading word, floating sub-heading word, keyword heading word, organism supplementary concept word, protocol supplementary concept word, rare disease supplementary concept word, unique identifier, synonyms, population supplementary concept word, anatomy supplementary concept word]	1242718
height.mp. [mp=title, book title, abstract, original title, name of substance word, subject heading word, floating sub-heading word, keyword heading word, organism supplementary concept word, protocol supplementary concept word, rare disease supplementary concept word, unique identifier, synonyms, population supplementary concept word, anatomy supplementary concept word]	211784
maternal weight.mp. [mp=title, book title, abstract, original title, name of substance word, subject heading word, floating sub-heading word, keyword heading word, organism supplementary concept word, protocol supplementary concept word, rare disease supplementary concept word, unique identifier, synonyms, population supplementary concept word, anatomy supplementary concept word]	3445
maternal height.mp. [mp=title, book title, abstract, original title, name of substance word, subject heading word, floating sub-heading word, keyword	1193

heading word, organism supplementary concept word, protocol supplementary concept word, rare disease supplementary concept word, unique identifier, synonyms, population supplementary concept word, anatomy supplementary concept word]	
Inflammation/	209212
Obesity/	216982
Obesity, Maternal/	651
Diabetes Mellitus, Type 2/	171839
Body mass index/	149307
((or/1-12) and (or/13-37) and (or/38-63)) or ((or/1-12) and (or/13-37) and (or/64-100))	9416
101 not (exp animals/ not humans.sh.)	4465

2. Appendix B: Modified Downs and Black Checklist for Measuring Study Quality

QUALITY ASSESSMENT QUESTIONS	SCORE	COMMENTS
REVIEWER’S INITIALS		
STUDY AUTHOR(S)		
YEAR		
REPORTING	Score (yes 1 / no 0)	
1. Is the hypothesis/aim/objective of the study clearly described?		
2. Are the main outcomes to be measured clearly described in the introduction or methods section?		
3. Are the characteristics of the participants include ed in the study clearly described?		
4. Are the interventions of interest clearly described?		
	Score (yes 2 / partially 1 / no 0)	
5. Are the distributions of principal confounders in each group of subjects to be compared clearly described? List of potential confounders.		
	Score yes 1 / no 0)	
6. Are the main findings of the study clearly described?		
7. Does the study provide estimates of the random variability (e.g. standard error, standard deviation, confidence intervals, inter-quartile range) in the data for the main outcomes?		
8. Have all important adverse events that may be a consequence of the intervention been reported?		
9. Have the characteristics of participants lost to follow-up been described?		

10. Have actual probability values been reported (e.g. 0.035 rather than <0.05) for the main outcomes except where the probability value is less than 0.001? If only actual values for non-significant results (i.e. 0.06) reported the question should be answered no.		
**EXTERNAL VALIDITY**	**Score (yes 1 / no 0 / unable to determine 0 / not applicable)**	
11. Were the participants asked to participate in the study representative of the entire population from which they were recruited?		
12. Were those participants who were prepared to participate representative of the entire population from which they were recruited?		
13. Were the staff, places, and facilities where the patients were treated, representative of the treatment the majority of patients receive?		
**INTERNAL VALIDITY – BIAS**		
**14.** Was an attempt made to blind study subjects to the intervention they have received?		
15. Was an attempt made to blind those measuring the outcomes of the intervention		
16. If any of the results of the study were based on "data dredging", was this made clear?		
17. In trials and cohort studies, do the analyses adjust for different lengths of follow-up of patients, or in case-control studies, is the time period between the intervention and outcome the same for cases and controls?		
18. Were the statistical tests used to assess the main outcomes appropriate?		
19. Was compliance with the intervention/s reliable?		

20. Were the main outcome measures used accurate (valid and reliable)?		
**INTERNAL VALIDITY - CONFOUNDING (SELECTION BIAS)**		
**21.** Were the participants in different intervention groups (trials and cohort studies) or were the cases and controls (case-control studies) recruited from the same population?		
22. Were study participants in different intervention groups (trials & cohort studies) or were the cases and controls (case control studies) recruited over the same period of time?		
23. Were study subjects randomised to intervention groups in trials?		
24. Was the randomised intervention assignment concealed from both patients and health care staff until recruitment was complete and irrevocable?		
25. Was there adequate adjustment for confounding in the analyses from which the main findings were drawn?		
26. Were losses of participants to follow-up taken into account?		
**POWER**	**Score (yes, one measure 1, no 0)**	
**27.** Did the study mention having conducted a power calculation to determine the required sample size?		
**TOTAL SCORE**	**/28**	

Appendix C: Quality Assessment

Study (First author surname year)	Quality scores (value)						
	Reporting	External validity	Internal validity - Bias	Internal validity - Confounding	Power	Total (score)	Rating
Brunner 2014	7	0	5	1	0	13	Low
Cagiran-Yilmaz, 2021	6	1	4	3	0	14	Low
Chan, 2017	7	3	5	2	1	18	Fair
Choi, 2022	7	2	5	2	0	16	Fair
Christensen, 2022	9	1	5	2	0	16	Fair
Cortes-Macias, 2023	6	1	4	2	0	13	Low
De-Luca, 2016	6	2	5	2	0	15	Fair
Ellsworth, 2020	7	2	5	2	0	16	Fair
Enstad, 2021	6	0	5	2	0	13	Low
Fields, 2017	6	1	5	2	0	14	Low
Gridneva, 2018	6	1	5	2	1	15	Fair
Khodabakshi, 2018	6	1	4	2	0	13	Low
Kunganathan, 2017	6	1	5	2	1	15	Fair
Larsonn-Meyer, 2021	6	2	5	2	1	16	Fair
Larsson, 2018	5	1	5	0	0	11	Low
Leghi, 2021	6	0	5	2	0	13	Low
Miralles, 2006	5	1	4	2	0	12	Low
Nuss, 2019	6	3	5	2	0	16	Fair
Pundir, 2020	6	3	5	2	0	16	Fair
Pundir, 2019	7	0	4	2	0	13	Low
Rodel, 2022	4	0	5	2	1	12	Low
Romijn, 2021	7	2	5	3	0	17	Fair
SadrDadres, 2019	6	1	5	3	0	15	Fair
Savino, 2016	6	0	4	2	0	13	Low
Schneider-Worthington, 2021	5	1	5	3	1	15	Fair

Schuster, 2011	6	0	4	2	0	12	Low
Uysal, 2002	5	0	5	2	0	12	Low
Weyermann, 2007	7	3	5	4	0	19	Fair
Young, 2018	8	2	5	3	0	18	Fair
Young, 2017	6	0	5	2	0	13	Low
Yu, 2018	7	0	5	3	0	15	Fair
Zamanillo, 2019	5	1	5	2	0	13	Low
Zielinkska-Pukos, 2022	7	1	5	3	0	16	Fair

Appendix C: Results from the quality assessment using the modified Downs and Black checklist. A quality score of ≥ 26 was considered excellent, scores between 20 – 25 were considered good, scores between 15 – 19 were considered fair, and scores ≤ 14 were considered low.
